# Targeting Histamine and Histamine Receptors for Memory Regulation: An Emotional Perspective

**DOI:** 10.2174/1570159X22666240128003108

**Published:** 2024-01-29

**Authors:** Zhuowen Fang, Jiahui Chen, Yanrong Zheng, Zhong Chen

**Affiliations:** 1 Institute of Pharmacology & Toxicology, NHC and CAMS Key Laboratory of Medical Neurobiology, College of Pharmaceutical Sciences, Zhejiang University, Hangzhou 310058, China;; 2 Key Laboratory of Neuropharmacology and Translational Medicine of Zhejiang Province, School of Pharmaceutical Sciences, Zhejiang Chinese Medical University, Hangzhou 310053, China

**Keywords:** Histamine, histamine receptor, emotional memory, attention, stress, memory

## Abstract

Histamine has long been accepted as a pro-cognitive agent. However, lines of evidence have suggested that the roles of histamine in learning and memory processes are much more complex than previously thought. When explained by the spatial perspectives, there are many contradictory results. However, using emotional memory perspectives, we suspect that the histaminergic system may interplay with stress, reward inhibition, and attention to modulate emotional memory formation. The functional diversity of histamine makes it a viable target for clinical management of neuropsychiatric disorders. Here, we update the current knowledge about the functions of histamine in emotional memory and summarize the underlying molecular and neural circuit mechanisms. Finally, we review the main clinical studies about the impacts of histamine-related compounds on memory and discuss insights into future research on the roles of histamine in emotional memory. Despite the recent progress in histamine research, the histaminergic emotional memory circuits are poorly understood, and it is also worth verifying the functions of histamine receptors in a more spatiotemporally specific manner.

## 1 INTRODUCTION

Memory is the retention of past experiences and information that may have an impact on an individual's future behavior [1]. The capacity to save and retrieve knowledge is one of the mammals' most intriguing abilities. As a trace of past experiences stored in the brain, memory is unique to each individual. However, memories are not formed instantly [2]; the processes of memory formation involve widely distributed regions of the brain and are accompanied by changes in molecular, synaptic and cellular levels [3]. Memory consolidation, a time-locked cellular storage process that is triggered by experience, occurs in several brain regions involved in the memory form being presented. Subsequently, memories can be successfully retrieved when subjects encounter specific contexts or cues related to previously learned memories [4]. Memory deficiency is common during aging and also the typical symptom of diverse disorders, including Alzheimer's disease (AD), vascular dementia, Parkinson's disease (PD) and so on [5]. Nonetheless, methods that can efficiently elucidate memory regulation are still insufficient.

Many factors can influence memory strength, among which emotion is one of the most important parts. William James forecasts the effect of emotion on memory in 1890 [6] and there is extensive evidence indicating that emotion may contribute to the establishment of the memory process which may influence the retention periods and strength of memories [7]. Many neuropsychiatric disorders are characterized by the coexistence of cognitive and emotional-behavioral deficits, such as epilepsy, Alzheimer’s disease, autism, schizophrenia, and depression [8, 9]. Exposure to trauma which is negative emotional arousal such as the violence of both nature and people, through earthquakes, floods, childhood abuse, *etc.* can cause post-traumatic stress disorder (PTSD) [10]. Subjects can easily develop a drug addiction in situations where they are highly rewarded, sharing similarities with neural plasticity linked to natural reward learning and memory [11]. There is still a need for an efficient treatment for emotional memory impairments. The pharmacological treatment for PTSD, for example, can only reduce the symptoms of anxiety and depression [12]. Existing psychotherapies featuring the utilization of cognitive principles related to emotional memory to produce therapeutic effects by reactivating old memories were more efficient. They engaged in new emotional experiences but NOT traumatic ones, at the same time reinforced the integrated memory structure [13]. The potential for emotional impacts to selectively enhance or, conversely, selectively inhibit certain forms of memory has significant clinical ramifications and may change how we approach psychological illness.

Histamine is a monoamine neurotransmitter and neuromodulator in the central nervous system (CNS), regulating feeding, drinking, sleep-wake cycle and cognitive functions such as attention, learning, and memory [14]. Histamine has long been accepted as an important modulator in spatial memory. However, there are still a lot of inconsistent findings about the function of histamine in spatial memory (*e.g*. both histamine and histaminergic H3R agonists RAMH or imepip can improve memory retention) [15]. From another perspective, histamine is also an important responsive neuromodulator to stress, anxiety or reward-related signals. It can be activated by some of stressors and is generally tacked as a reward inhibitor. This property of histamine can be indicated as a sensor of emotional salience or valence. Meanwhile, histamine can impair learning and memory related to rewards, enhance fear memory under mild stress, and decrease fear memory under severe stress. Histamine can also have a complex impact on stress-associated elevated plus maze (EPM) memory. Finally, histamine may also take part in attention modulation. Of particular interest in understanding how histamine modulates different emotional memories, and whether through valence encoding or salience encoding, we categorize different memories according to their emotional valence. As a result, we will use this kind of system to describe the relationship between histamine and emotional memory in this review.

## 2 EMOTION AND EMOTIONAL MEMORY

Cognitive neuroscientists are now focusing on how emotional events are stored in the human brain for learning and memory. These findings help to bridge the gap between clinical diseases and animal models and are beginning to explain how emotional memory networks are structured on a systems level. The two most well-known theories are the “two-factor theory of emotion” and “the two-dimensional theory of emotion”, which are suggested by emotion theorists as explanations for how emotions are processed. Based on these theories, emotions can be classified on two scales: salience, which ranges from low to high, and valence, which ranges from negative (unpleasant) to positive (pleasant) [16]. Salience is the ability of a stimulus to direct attention that engages decision-making processes and elicits an incentive motivational response. Additionally, a stimulus' salience is influenced by both its physical and behavioral relevance, such as a relative quality that is dependent on prior knowledge or the present state of homeostasis [17]. Valence is a defining feature of a stimulus that endows it with the capacity to elicit a positive or negative affective, emotional state or response. An implication of portraying the two-dimensional theory of emotion in this manner is that emotions with the most extreme positive and negative valence generate the highest levels of arousal. In recent years, using behavioral techniques combined with *in vivo* single-cell calcium or electrophysiological recordings, researchers have identified systems that encode emotional salience and valence separately and coherently. For emotional valence encoding, brain regions, including the amygdala, sensory cortices, striatum paraventricular thalamus (PVT) *etc.* [18-20] that respond to different emotional valences and serve to encode emotional valence; brain regions such as the amygdala, PVT, basal forebrain, and prefrontal cortex that respond to varying intensities of stimuli of the same valence in a salience-dependent manner are used to encode emotional salience [21-23]. Apart from fast neurotransmitter systems, neuromodulator systems (corticosteroid, noradrenergic, endocannabinoids, dopamine *etc.*) can provide a feedback signal with a slower temporal scale by acting in G-protein-coupled receptors and second messengers to guide plasticity within other circuits. It is critical to focus on how emotion affects memory according to the salience and valence and the relationship between the two dimensions.

**Fig. (1):**
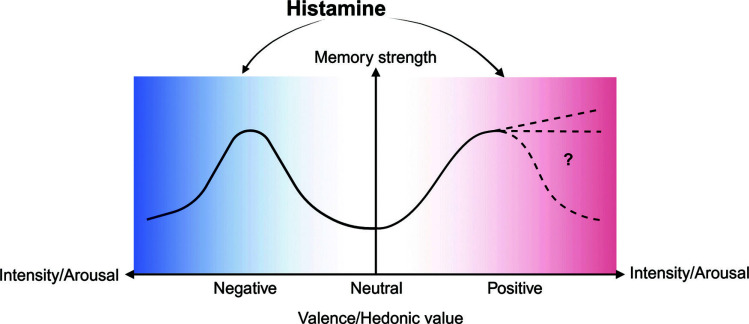
A schematic overview of the function of histamine in memory. Emotional memory strength can be influenced by both emotion valence and salience. For negative emotion, with the increasing emotional salience, memory performance increases with physiological or mental arousal, but only up to a point. When arousal levels become too high, performance decreases due to excessive stress coupling with too many neurotransmitters, such as noradrenaline and dopamine, which impair memory under high concentration. For positive emotion, with the increasing emotional salience, memory performance increases, and whether it can reach a point or a plateau remains to be elucidated. Histamine may be involved in both emotional valence and salience processing to influence emotional memory formation.

“Emotional memory” is the memory of a past event that elicited an emotional response. The terms “emotional memory” and “memory for a past emotional state,” among others, should not be confused with this definition. Throughout this article, we will stick with the more commonly used term “emotional memory” and its branches, “negative memory” and “positive memory,” to refer to events that initially elicited a negative or positive affective response [24]. Positive and negative emotional stimuli can both strengthen memory compared to neutral stimuli. In the cognitive neuroscience field, numerous theories and processes that unfold during the experience of a negative event have been proposed as described, and the seconds, minutes, and hours that follow can make these memories durable (Fig. **1**).

Emotional arousal stimuli boost neural competition during the memory encoding process, helping emotional stimuli “win” additional processing while making neutral stimuli “lose” processing resources. This is manifested as narrowing attention, which especially enhances memory associated with the central details and degrades memory associated with background details. An arousal-biased competition (ABC) model was suggested as the process [25]. Meanwhile, Talmi’s mediation model focused on emotional memory encoding proposes that emotional experiences benefit from boosted attention, elaboration, and organizational processes implemented at encoding [26]. During consolidation processes, the modulatory influence of emotional arousal *via* the amygdala, specifically on memory regions such as the hippocampus. To explain this mechanism, there are primarily two theories. Firstly, extensive research demonstrated that the arousal associated with a (negative) emotional event triggered stress hormones that set off a cascade of processes, resulting in upregulation of amygdala function and increasing amygdala-hippocampal connectivity and synergy of action [27]. These processes can be concluded as a modulation model: amygdala activity was enhanced during the successful encoding of negative content, and its relation to memory often was related to its interactions with the hippocampus [28]. Second, an emotional binding model proposes that the amygdala functions separately to link items to their emotional sensitivity, adding emotional memory by claiming that the hippocampus helps to connect contextual data into an episodic memory representation [29]. Within the last few years, there has been a more direct focus on the role of retrieval processes in giving negative emotional memories their power. One is the emotional Context Maintenance and Retrieval model (eCMR): memory bound to emotional as well as temporal context and emotional context maintain robust even after longer delays [30]. The second is called Negative Emotional Valence Enhances Recapitulation (NEVER Forget). When memories are unpleasant, there is a higher chance that the brain will rearrange itself during retrieval to have a better encoding-to-retrieval overlap, which is associated with memory success and confidence [3]. While emotional memory retrieval is typically assessed as a final outcome, it can alternatively provide a fresh chance for encoding and stimulate storage processes [31]. Besides, the neuromodulatory model indicates that an emotional arousal state can cause the release of stress hormones like epinephrine and glucocorticoids, which might interact with the amygdala. While noradrenaline and dopamine interact with the prefrontal cortex (PFC), stress hormones may have an “inverted U-shaped” influence on the brain physiology and cognition due to different affinity of receptors interacting with final concentration, resulting in different activity patterns [32]. As a consequence of higher emotional salience accompanied with more stress hormones release, emotional memory can be improved by these stress influence and then reach a peak followed by memory disruption.

Positive memories derive strength from many of the same factors that give negative memories strength: longevity and accessibility. However, positive and negative experiences do not always have similar memory properties (Table **1**). (1) Positive memories may not show the same automaticity of retrieval mechanisms as negative events; positive events from our personal past are more likely to be recalled than negative events and may be recalled unintentionally [24]. (2) It is rich in associations, which may increase the probability that retrieving one positive memory will trigger another. Memories of positive experiences may include more of the conceptual framework or gist and are more likely to include contextual associations than memories of negative experiences. Memories of positive experiences may also be associated with the general knowledge that an event has occurred rather than being able to recall specific details [3]. (3) Positive memories show a shallow forgetting curve for the event and affect. In a phenomenon known as Fading Affect Bias (FAB), the affect of negative memories tends to fade over time, whereas the effect of positive memories tends to remain strong [33]. (4) It is possible that positive *versus* negative memories may be different in amygdala-binding and hippocampal-binding systems coordination. For positive memories, there is both amygdala-binding and hippocampal-binding [34, 35]. At the same time, negative memories may be associated with enhanced amygdala-binding and reduced hippocampal-binding [35]. (5) In contrast to negative memories, which are more likely to engage sensory regions linked with emotional memory networks, the acquisition and retrieval of positive information tends to be associated with higher activity in prefrontal regions [36].

The amygdala plays a vital role in emotional learning and memory consolidation by acting as the primary brain structure that directly mediates these processes while also facilitating related functions in other regions, such as the hippocampal and prefrontal cortices [37]. The evidence so far seems to support that there exist both valence- and salience-encoding neurons in the amygdala. By labeling aversive (shock, air puff *etc*.) and appetitive (sucrose solution *etc*.) stimulus-activated neurons in the same mice, results indicate that neurons in the amygdala can encode negative and positive valence. As for salience encoding neurons analysis, there are two types of methods: firstly, provoke animals by using stimuli with different levels of salience and record calcium activity or electrophysiology of neurons. In the meantime, neurons are taken as salience encoding when the change of activity is related to the salience of the stimuli; secondly, when modulating specific neuron activity during the behavioral test, the degree of the neuron’s activation is related to the animal’s behavioral outcome, and then these neurons are also salience encoding neurons [38, 39].

## 3 HISTAMINERGIC SIGNALING IN THE BRAIN

Histaminergic neuronal somata are grouped in five clusters E1-E5 within the tuberomammillary nucleus in the hypothalamus (TMN) and project to a wide range of regions in the brain [40]. Brain histamine is synthesized from L-histidine by histidine decarboxylase (HDC) [41]. Inactivation of histamine in the brain is through the only known pathway by the action of histamine N-methyltransferase (HNMT) transfers a methyl group to histamine and is oxidized by monoamine oxidase B (MAOB) [42, 43]. Histaminergic neurons establish synapses predominantly in the mesencephalic trigeminal nucleus and project to almost everywhere else in varicosities format, which contains synaptic vesicles [44-46]. The histaminergic network may play a broad modulatory function of the ability to release histamine at non-synaptic sites and the lack of a high-affinity re-uptake system [43, 47]. Brain histamine has also been reported to be produced by mast cells [48], microglia [49], and microvascular endothelial cells [50], but the function of this sort of histamine has not been elucidated. Histamine functions by binding to histamine receptors. Four G-protein coupled histamine receptors have been found (H1, H2, H3, H4), and the H1-3 receptors have received more attention in the brain. H1 and H2 receptors mainly expressed postsynaptic existing in neurons and glial cells. H3Rs are mostly presented in presynaptic as autoreceptors controlling the release of histamine or heteroreceptors regulating other neurotransmitters’ release. Research on postsynaptic H3Rs is still in its infancy. The results obtained provide a very limited understanding of the role of H3R in-memory processing [51]. H4Rs have recently been identified and mainly focus on their role in the inflammatory processes [52].

## 4 CONTRADICTORY ROLES OF HISTAMINERGIC SYSTEM MEMORY: FROM A SPATIAL PERSPECTIVE

Brain histamine has important modulatory functions on memory processes (Table **2**). The first paper was published in 1986, suggesting this by Almeida and Izquierdo [53]. They claimed that histamine injections given intracerebroventricularly (i.c.v.) after training improved rats' ability to retain inhibitory avoidance memory. Histamine's involvement in memory and learning has been examined in several processes utilizing gene-knockout, pharmacological, and a few chemogenetic investigations. Most studies indicate that histamine facilitates memory retention, but some other reports propose memory is inhibited depending on the tasks and brain regions, so the overall picture is still unclear [15, 54].

Histamine injection *via* the i.c.v. route can enhance memory retention in both stepdown inhibitory avoidance tasks [55] and active avoidance response [56]. In the passive avoidance learning test, rodents learn to suppress their natural tendency to seek out dark areas or step-down areas which are paired with mild foot shock. The longer the time to step through or step down means better memory. In active avoidance, the animal learns to avoid shock which is coupled with CS, by moving from one side of the apparatus to the other. In the Barnes maze, early research found that i.c.v. or intraperitoneal (i.p.) injection of histamine can ameliorate the amnesia in MK-801 or αFMH induced memory deficit rats [57]. Furthermore, everyday systemic administration of H3R antagonists can ameliorate the adverse effects on chronic restrain stress model, MK-801, scopolamine, phencyclidine (PCP) or α FMH induced memory deficit models [57-61]. H3R agonists have the opposite effect, similarly [58, 59]. Most importantly, i.c.v. of histamine and i.p. histidine improved memory deficits in a rat hippocampal lesions-induced active avoidance paradigm [62]. A basolateral amygdala infusion of histamine (0.5 mA, 2 s) increased memory consolidation of inhibitory avoidance [15]. Antagonizing H1R systemically can increase reference memory and working memory errors in the Barnes maze [57, 63]. H1R antagonists administered intrahippocampally can counteract the procognitive effects of thioperamide, an H3R antagonist, on working memory in MK-801-induced amnesia. When infused into the CA1 region of the dorsal hippocampus immediately after training in an inhibitory avoidance task or contextual fear conditioning, histamine or H2R agonist can enhance memory consolidation (without influence anxiety state, 2 s, 0.5 mA) [64]. It has also been reported that blocking the H1R or H2R in the amygdala or hippocampus impaired latency and efficiency of learning-cued fear memory (0.025 mA) [65]. In a dose-dependent way, intra-CA1 infusion of histamine following non-reinforced retrieval (2 s, 0.8 mA) increased consolidation of inhibitory avoidance extinction [66]. The inhibitory avoidance memory deficit induced in rats by early postnatal maternal deprivation (0.4 mA, 2 s) was reversed by histamine [54]. Mice lacking histidine decarboxylase (HDC-KO) were incapable of producing histamine and experienced difficulty in learning a temporal sequence that involved introducing new objects into a familiar setting in succession [67]. Both H1R knockout (H1R-KO) and H2R knockout (H2R-KO) mice showed memory deficits in the radial maze, while H3R knockout (H3R-KO) mice have better memory retention [68].

Nevertheless, conflicting findings exist. For instance, rats on a histidine-deficient diet exhibited decreased histamine levels in the hippocampus and enhanced performance on an eight-arm radial maze [69]. Conversely, administration of histamine *via* intracerebroventricular injection immediately after training affected retention of a one-trial step-through passive avoidance task measured 24 hours post-training in rats (at 1.5 mA) [70]. Activating H3R within the BLA by post-training microinfusion of histaminergic H3R agonist R-alpha-methylhistamine (RAMH) and immepip showed stronger memory for the context-footshock association (7*1 s,1 mA shock) [71]. Activating H3R within the dorsal hippocampus by an H3R agonist can also improve memory consolidation in contextual fear conditioning (7*1 s,1 mA shock) [72]. Additionally, administration of the H1R antagonist chlorpheniramine directly into the ventricles enhanced water-maze performance in older rats [73], as well as improved retention performance in a one-trial step-through passive avoidance task when administered immediately after training and tested after a 24-hour retention interval [70]. When given intracerebroventricularly, the H2R agonist 4-methylhistamine reduced the step-through latency in a mouse step-through passive avoidance task retention trial [74]. HDC-KO exhibited improved contextual fear from 1-14 days, and cued fear was also improved 2 days after training, accompanied by increased LTP and presynaptic glutamate release in hippocampal CA1 (2 s, 1 mA) [75]. Additionally, it has been observed that HDC-KO mice performed better on concealed and cued platform tasks in the water maze [67]. There is also one report on improved auditory and contextual fear-conditioning in H1R-KO mice (2 s, 0.3 mA) [76].

**Fig. (2):**
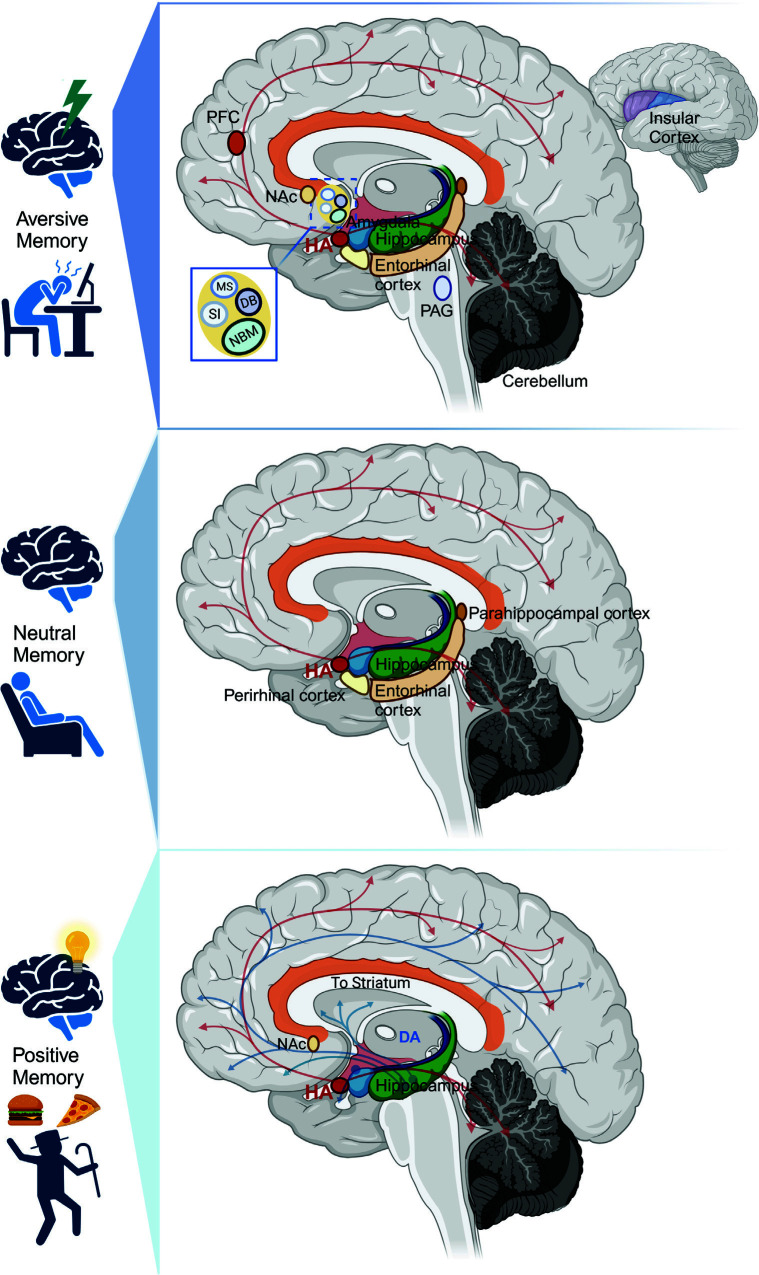
A schematic diagram of the histaminergic network underlying aversive memory, neutral memory and positive memory. Histamine is required for aversive memory, which includes fear memory, stress memory and taste aversion memory. The histaminergic neuron system innervates with amygdala, hippocampus, insular cortex and basal forebrain *etc*. to modulate aversive memory. As for neutral memory, the histaminergic projections to the perirhinal cortex, hippocampus *etc*. might engage in novel object recognition. Finally, the histaminergic neurons regulate positive memory through the reward system and hippocampus. [100, 103].

From the above analysis, it is easy to see some of the results support a facilitative effect of histamine on spatial or working memory, but in some different experimental details, similar administration principles can produce the opposite experimental results. Thus, we compare the details and parameters of the most studied fear memory, which contain at least a shock (*i.e*., inhibitory avoidance, active avoidance, contextual or cued fear conditioning). Surprisingly, we find that histamine often displays pro-cognitive effects in experiments that constitute low current intensity (lower than 1 mA) [15, 54, 64, 66] while playing a detrimental role when the shock was greater than or equal to 1mA in most situations [71, 72, 75]. It seems that the role of histamine in memory may be dependent on the emotional properties of the event. Histamine may engage partly in both encoding emotionally related information and episodic information to modulate emotional memory. Therefore, we’ll discuss the role of histamine in memory according to an emotional perspective in the following part of this review (Fig. **2**).

## 5 THE ROLE OF HISTAMINE IN MEMORY: FROM THE EMOTIONAL PERSPECTIVE

### 5.1 Histamine and Emotional State

#### 5.1.1 Stress

Stress has a strong connection with histamine release. For example, using the microdialysis technique, a large increase in histamine release was observed in the TMN of rats under handling stress for 15 min [77]. Using c-Fos immunocytochemistry combined with *in situ* hybridizations of HDC mRNA, researchers find that different subregions of histaminergic neurons act differently when facing different stress situations: restraint, insulin-induced hypoglycaemia and foot shock can cause specific activation of histaminergic E4 and E5 subgroup; some neurons of the E1, E2, E3 were activated after restraint stress and no c-Fos change in either hyperosmotic stimulus or injection of bacterial lipopolysaccharide [78]. Additionally, histamine can interact with the most significant system for producing stress hormones, the hypothalamic-pituitary-adrenal (HPA) axis. In the social isolation model, stimulation of central H1R can cause hyperresponsiveness of the HPA system through changes in the efficacy of H1R but no changes in the brain monoamine levels [79]. There is evidence indicating that hypothalamic histamine and histamine receptors may be involved in the HPA stimulation *via* the muscarinic system [80] or *via* constitutive cyclooxygenase and endogenous prostaglandins [81] and endogenous NO [82]. On the other hand, crowding stress can abolish the rise in hypothalamic histamine induced by beta- and alpha-2-adrenergic agonists [83]. Histamine plays a crucial role in facilitating stress-induced surges of neuroendocrine hormones such as ACTH, β-endocrine, and AVP from the pituitary gland, and it also regulates the activity of aminergic systems associated with stress, including neurons that contain serotonin, norepinephrine, dopamine, and acetylcholine [84]. The activation of the HPA axis through CRH release is caused by histamine injections in the PVN [85]. Thus, we can conclude that histamine is not only sensitive to stress state but also has complex interaction with stress-related hormones. In addition, a pharmacodynamic dose curve that follows an inverted U-shape can be observed when the histaminergic system is manipulated using either H3R antagonists or H2R agonists in tasks related to social memory, Y-maze working memory, novel object recognition memory, and inhibitory avoidance memory [86]. To put it simply, this phenomenon can be explained as follows: normal level of histamine keeps animals under an alert or arousal state with optimal memory function while too much or too little histamine are all deleterious. However, what induces histaminergic activation under stress is still unknown. Which upstream of histaminergic neurons or what kind of direct or indirect molecular receptors are participating in these processes remain murky. Except from central neural histamine, there’s evidence showing that under stress circumstances mast cell-derived histamine may induce abnormal neural signaling by the activation of enteric nervous system [87, 88]. Additionally, mast cells located in the leptomeninges and brain capillaries release both histamine and CRH in response to systemic stress, highlighting the complex interplay between the nervous and immune system as well as histamine and CRH [85].

#### 5.1.2 Anxiety and Aversion

Histamine may be a danger response signal that promotes anxiety, as suggested by pharmacological and genetic studies in rodents [89]. Lesions in the TMN reduce anxiety [90]. L-histidine peripheral injection dramatically reduced the amount of time that subjects spent exploring in open arms, and such effects were diminished by the H1R antagonist pyrilamine [91]. Consistently, microinfusing H1R antihistamine chlorpheniramine or H2R antagonist ranitidine into the nucleus basalis magnocellularis produces anxiolytic activity [91]. Coincidentally, H3R antagonists raised anxiety levels in wild-type mice but not in Apoe-/- mice, supporting a function for apoE in H3R signaling [92]. When combined with zolantidine H2R blockade, thioperamide-induced histamine increases become anxiogenic [93]. HDC-deficit mice that lack the ability to synthesize histamine show increased measures of anxiety, and histamine can ameliorate anxiety through activation of heteroreceptors H3R in NAc or modulate the neural circuits in NAc, which holds a key position in motivation, emotion and cognition [94-96]. Considering these findings collectively, it is possible that histamine plays a multifaceted function in anxiety and the reinforcement of anxiety-related behaviors.

#### 5.1.3 Pleasure and Reward

Although it is still debatable [97], it is believed that brain histamine has a mostly inhibiting impact on primary reward [98]. The data indicates that central histamine opposes the reward and reinforcement mechanisms that are controlled by the mesolimbic dopamine system [99]. Moreover, histamine could contribute to morphine addiction, as evidenced by a considerable reduction in endogenous brain histamine levels following long-term exposure to morphine and sudden withdrawal [100]. In addition, rats that have lesions in the TMN exhibit reduced thresholds for rewarding electrical brain stimulation in the lateral hypothalamus [101]. However, blocking histamine synthesis unilaterally by injection of αFMH into TMN surprisingly reduces the rate of ipsihemispheric lateral hypothalamic self-stimulation, which is opposite to the behavioral effects of TMN lesions [102]. The findings imply that histamine predominantly functions as a suppressant of the reward and reinforcement mechanism in the brain, consequently impacting learning and memory formation dependent on reinforcement.

### 5.2 Histamine in Aversive Memory

#### 5.2.1 Histamine in stress-based Learning

The elevated plus-maze (EPM) is a model used in a repeated measurements paradigm to identify the impact of stress situations on memory. Although the EPM is commonly used to assess animal anxiety, repetitive testing can serve as a gauge for acquisition and memory retention, evidenced by experience-driven behavioral alterations, whereby rats demonstrate diminished exploratory conduct during subsequent sessions [103]. Gianlorenço *et al.* used this special behavioral test to explore mice’s memory under stress. They found that histamine or histidine both impaired emotional memory in EPM no matter whether systemic administered or applied in the amygdala or cerebellar vermis [104, 105]. However, blockade of H1R in the dorsal hippocampus and the highest dose of H1R antagonist chlorpheniramine in BLA can also impair emotional memory in EPM [106]. These contradictory results on the role of histamine in stress memory may coordinate with the complex role of histamine in anxiety and indicate that histamine may interplay with perceiving anxiety and storing anxiety-related memory in distinct brain regions and circuits.

#### 5.2.2 Histamine in Fear-related Memory

Numerous early studies have focused on fear (shock)-related memory, as mentioned above, and the results display polarized results: some of them find activating the central histaminergic system can enhance memory retention, while others find the opposite. We conclude these results as histamine displays a pro-cognitive role under mild foot shock intensity (<1 mA), which elicits medial emotional intensity, while under higher foot shock intensity condition accompanied by extremely higher emotional salience and activation of stress neuromodulators. As aforementioned, histamine can activate the HPA axis and foot shock stress may activate the histaminergic system; it seems that extremely high foot shock intensity may overstimulate the histaminergic system, therefore accelerating the overexpression of stress hormones. Overly strong or aberrant stress effects on cognitive processing can become maladaptive [107]. Thus, under appropriate shock intensity, histamine displays a pro-cognitive effect, and these things are reversed when the stress intensity is higher than the peak threshold of the inverted U-shaped curve [32]. Nowadays, recent research is more inclined to choose an appropriate shock intensity which is around 0.3-0.8mA and explore details about the relationships between the histaminergic neuron system and fear memory under these conditions.

Intra-lateral ventricles administration of αFMH (a suicide inhibitor of histidine decarboxylase, used for histamine depletion *in vivo*) can impair long-term IA memory (inter-trial interval, ITI = 24 hours-7 days) but keep short-term memory intact (ITI=2 hours) [108, 109]. This long-term memory-specific role indicates histamine may play a role in consolidation rather than memory encoding. Furthermore, betahistine, a modest H1R agonist and a powerful H3R antagonist, systematically administered into PTZ kindling animals can ameliorate the inhibitory avoidance retention memory deficit [110]. In accordance with this, systemic administration of H3R (auto-receptor) antagonists ABT-288 or E177 can increase histamine release and improve fear memory retention in normal animals or rescue the deficits in chronic constrain stress model, MK-801 (an uncompetitive antagonist of NMDAR) induced amnesia or PTZ-induced memory deficits model [111, 112]. H3R agonist RAMH or imetit may inhibit histamine release and inhibit IA memory [113]. In addition to its role in memory consolidation, histamine is also involved in the retrieval of fear memories. Antagonizing H3R before the test can generally promote memory or attenuate the impairment in the MK-801-induced deficit or isoflurane-associated cognitive deficit model [111, 114-117]. Also, there’s research showing that antagonizing H3R can rescue memory in chronic constrain stress [118]. As for fear memory reconsolidation, findings suggest that H3R antagonists can reverse the deficit in MK-801-induced amnesia mice but have no effect on control mice [119]. Thus, the histamine system is important for retaining fear memory successfully.

More specifically, microinjection of histamine into the amygdala or ventral hippocampus can promote IA memory, indicating amygdala or ventral hippocampus may be important for the role of histamine in IA, which plays different roles [120]. Histamine can antagonize the amnestic effect of αFMH microinjected into BLA in the early IA consolidation period (post-training 0-110 min), whereas having no effect injecting 110 min after training. By comparison, histamine has a longer active time window (post-training 0-6 hours) in CA1 to rescue αFMH-induced amnesia. Furthermore, histamine acting at CA1, BLA or cerebellar vermis can also promote IA consolidation in healthy mice [15, 109]. And HNMT inhibitor SKF91488 can mimic histamine’s IA consolidation-promoting effect in CA1 and BLA [15, 64, 121]. In the fear memory consolidation process, H2R antagonist ranitidine and cimetidine can block the enhancive effect of histamine in IA consolidation when administered in CA1 and cerebellar vermis [64, 121], and zolantidine can antagonize the procognitive effect of thioperamide in NBM [15, 122]. However, H1R antagonists don’t have such effects [64, 121, 122]. Injecting H2R antagonists ranitidine or cimetidine directly into the cerebellar vermis and septum, as opposed to CA1 immediately following training, can impair IA retention. Conversely, directly activating H2R in NBM, CA1 and septum can enhance fear memory retention. This indicates the possible involvement of endogenous cerebellar vermis and septum, rather than CA1 histamine H2R, in the consolidation of IA memory during the analyzed consolidation period [122-124]. On the contrary, directly antagonizing H1R in NBM, CA1 and cerebellar vermis did not influence fear memory consolidation [64, 122, 124]. Local infusions of H3R antagonists into NBM and septum except CA1 can facilitate retention or reverse MK801-induced amnesia in fear memory, while activating H3R will get the opposite results [122, 123]. It is believed that in most situations H3R activation act as autoreceptors at presynaptic to inhibit histamine release and synthesis. However, H3R can also play a role as a heteroreceptor at presynaptic to influence other synaptic transmission systems or locate at postsynapses. For example, intra-BLA injection of H3R antagonist impaired the fear memory consolidation both in IA and fear conditioning [125] and activating H3R in BLA during fear memory consolidation enhances the memory retention which is opposite to the brain-wide effect of histamine [15, 119]. These effects might be explained by acetylcholine modulation in the BLA. While activating muscarinic receptors in the amygdala is essential for fear memory consolidation [126], intra-BLA administration of H3R antagonists or agonists can reduce or enhance acetylcholine release locally [71, 125]. Therefore, these results may indicate that histamine signaling is required for fear memory consolidation act with H2R but not H1R and may influence other neurotransmitter systems, such as the acetylcholine system.

#### 5.2.3 Histamine in Conditioned Taste Aversion

Conditioned taste aversion (CTA) is a reliable sort of learning that can be used to foretell the detrimental visceral effects of oral ingestion. It relies on selective associations between taste and visceral cues, and consequently, the taste becomes disliked and will be avoided in later presentations [127]. The administration of H1R agonists in the NBM can increase the Ach release in the cortex, including the insular cortex [128]. Additionally, painful taste memory development is hampered but not its retrieval when the H1R in the NBM is blocked, or the H3R in the insular cortex is activated [129].

### 5.3 Histamine in Neutral Emotional Memory

#### 5.3.1 Histamine in Novel Object Memory

Early efforts by the group of Blandina *et al.* (1996) found that intraperitoneal (i.p.) injection of H3R agonists imetit and RAMH can impair object recognition combined with reduced evoked release of cortical acetylcholine [113]. Different results have been reported when systemically administered H3R inverse agonists in delay-induced memory deficit or scopolamine (a muscarinic antagonist) -induced memory deficit mice. While some studies have indicated that blocking the H3R has no effect on these two forms of amnesia, others have discovered that it can prevent the deficit or have a U-shaped dose-dependent effect [59, 86, 130]. Thus, there’re many more details that need to be elucidated.

Administering H3R agonists before rather than after training has a negative impact on rats' object recognition memory performance. On the contrary, H3R antagonists can ameliorate scopolamine-induced amnesia both in memory encoding and consolidation processes but can’t exhibit any procognitive effects in normal animals. The former experiments, which use a 60 min inter-trial interval, indicate that H3R might participate in different stages of memory formation *via* different mechanisms [131]. When inter-trial intervals elongate to 24 hours, Trofimiuk *et al.* find that systemic administration of H3R antagonist ciproxifan before or after training can alleviate chronic constrain stress-induced amnesia but only post-training administration can improve memory in normal rats [118]. On the other hand, in a different study, the administration of the identical amount of H3R antagonist ciproxifan did not have any impact on the memory deficit induced by either delay (ITI = 24 hours) or scopolamine (ITI = 1 hour). Different experimental details may lead to varying outcomes. In the consolidation process, to be more specific, histamine activates in CA1 through both H1R and H2R in the early consolidation processes (post-training 0-120 min). Blockade of H1R and H2R in the efficient time window can impair NOR memory retention, but this effect disappears when administering drugs after training 360 min [132].

Apart from participating in memory consolidation, histamine also plays a role in memory retrieval processes. In a delay-induced memory deficit model (ITI=3 days, 1 week, 1 month), applying H3R antagonist thioperamide immediately before memory retrieval can make mice retrieve forgotten memory successfully. Furthermore, Nomura *et al.* found that the PRh is a critical region where histamine plays its role in novel object memory retrieval through H2R [133]. Consistently, blockade of H3R can also improve memory retrieval in chronic constrain stress, isoflurane-associated cognitive deficits or MK-801-induced memory deficits [59, 114, 115, 118]. These findings indicate the potential role of histamine in rivalry with amnesia or psychosis-accompanied memory deficits.

### 5.4 Histamine in Positive Memory

In the brain's reward and reinforcement system, histamine may, as noted above, perform a tonic inhibitory effect [99]. More crucially, disabling the suppression of brain reward and reinforcement systems by inhibition of histaminergic neurotransmission can improve learning and memory. The activation of the histaminergic system, for instance, can decrease the morphine-induced conditioned place preference (CPP). Meanwhile, the enhancement of the morphine-induced rewarding effect can be suppressed through the inhibition of histaminergic neurons, which may involve D1 receptors [134]. HDC-KO mice showed stronger ethanol or morphine-CPP, and this effect can be blocked by H3R antagonist [135, 136]. H3R antagonists, however, are unable to reduce alcohol reward in HDC-KO mice, indicating that histamine is necessary for the H3R-mediated suppression of alcohol-CPP [137]. H1R antagonists can cause a conditioned location preference in goldfish. The rats that received treatment with the H1R blocker in the caudal nucleus accumbens demonstrated increased rates of ipsihemispheric self-stimulation compared to the rostral group and also developed a conditioned place-preference [138]. These findings imply that histamine inhibits the reward and reinforcement system of the brain as well as learning- and memory-dependent processes.

## 6 HOW HISTAMINE WORKS ON EMOTIONAL MEMORY

### 6.1 Histamine in Emotional State Perception

The research has supported the idea that histamine inhibits the brain's reward and reinforcement system while also acting as a stress indicator by activating histamine in response to stress signals. However, histamine has a complex role in anxiety signaling. Besides histamine, monoamine neurotransmitters like serotine (5-HT) and dopamine (DA) have all been investigated in their diverse roles in emotional valence or salience responsiveness and distinct encoding subpopulations and networks. For example, Paquelet *et al.* find that dorsal raphe serotonin neurons are modulated during emotionally salient behaviors using highly correlated ensembles with mixed selectivity and biases in downstream connectivity [21]. Histaminergic neurons from distinct subpopulations display specific responsiveness to different stimuli such as restraint, insulin-induced hypoglycemia, or foot shock [78]. Thus, it is possible that distinct histaminergic neuron subpopulations may have the ability to response to different emotional valence and salience, which need to be further elucidated.

### 6.2 Histamine Interplay with Stress Modulators in Emotional Memory Retention

Within milliseconds after stressor onset, multiple stress mediators are released in waves and reach the brain at different time points. Each of the multiple stress mediators has its specific temporal profile of action on the brain, and the temporal windows of action may overlap, thus enabling synergistic actions between stress mediators. Stress mediators can change cell activity. For example, stress mediators have a quick and strong excitatory effect on particularly BLA neurons [139]. Moreover, stress-induced changes initially lead to prioritized attentional and appraisal processing of emotionally salient events, increase the reliance on well-established habits and routines, reduce distraction by stressor-irrelevant information, and promote the storage of information most relevant of the stressful encounter [107].

Generally speaking, stressors consist of dopamine, noradrenaline, serotonin, and the HPA axis [107]. In some brain regions, such as mPFC, either too little or too much stress mediators can markedly impair memory function [32]. Previous studies have indicated that histamine has responsiveness to specific stress, including foot shock. Coincidently, histamine seems to promote memory retention under mild shock intensity while impairing memory retention under quiet intense shock which may trigger the over-activation of stress mediators. In addition, the turnover rates of histamine in the diencephalon, nucleus accumbens, and striatum show an increase under conditions of stressful stimulation, such as exposure to electrical shocks or chronic restraint stress [77]. Histamine can also activate some of the stress hormones result in even worse memory retention abilities in this occasion. All these results indicate that histaminergic system can be activated under certain stressful situations and strengthen the stress effects through activate stress hormones. And this is one of the possible ways in which histamine regulated emotional memory.

### 6.3 Histamine in Attention Bias

It has long been proposed that neuronal histamine may serve as an alert signal in the brain when high attention or a strong wake-drive is needed, such as during exploration, self-defensive and learning. Enhanced histaminergic neurotransmission may help performance or sense of signals concerning internal or environmental dangers [140]. On the other hand, there is a suggestion that a slight decrease in levels of brain histamine could improve focus when tackling visuospatial tasks under stressful conditions [141]. Stress-induced changes initially lead to prioritized attentional and appraisal processing of emotionally salient events. Emotional salient stimuli can benefit from boosted and narrowed attention to specifically remember central details rather than peripheral details [25]. Thus, attention may be attracted by stressful stimuli and only under situations like lower histamine concentration can subjects spare their attention to some peripheral details related to escape from the shock maze. Therefore, histamine may take part in modulating attention towards emotional salience events. Notably, it is interesting to figure out whether histamine is required to guide attention to salient stimuli and support mnemonic operations that form initial memory traces or just engage in stress level modulation to influence attention distribution.

### 6.4 Histamine in Emotional Memory Formation

There are several steps involved in memory formation, including acquisition, consolidation, retrieval, and extinction. As aforementioned, histamine plays an important role in fear memory consolidation, which specifically works through H2R and H3R. Besides, there are several characteristics of histamine in the memory formation processes. Taking fear memory for example, modulation of the histaminergic system in BLA has a shorter time window compared with the hippocampus; modulating histamine in the hippocampus rather than BLA can influence memory retrieval. According to the modulation model and emotional binding model, the hippocampus and amygdala play a distinct role in emotional memory formation, and the hippocampus prefers to bind contextual details while the amygdala takes more emotional information. It is indicated that histamine may selectively play a role in integrating emotional information into contextual information by specific histamine receptors.

There are various mechanisms involved in the formation of emotional memories, such as synaptic plasticity, engrams, and network oscillations. Synaptic plasticity plays a crucial role in associative emotional learning and is believed to be a mechanism by which certain groups of neurons (engrams) are engaged during learning and reactivated during memory retrieval. Concurrently, synchronous oscillations in the theta and gamma frequency bands within the amygdala and interconnected brain regions have been observed during the consolidation and retrieval of emotional memories [142]. Ensembles of neurons that synchronize firing with each other and with afferent neurons located far away are produced by network oscillations. As a result, oscillations may have a significant impact on synaptic plasticity by accurately coordinating presynaptic and postsynaptic activity. Histamine is suggested to modulate excitatory synaptic transmission in both directions based on electrophysiological recordings conducted in the basolateral region of the amygdala [143]. Moreover, histamine can improve long-term potentiation (LTP) in the hippocampus *via* H1R, H2R, or H3R both *in vivo* and *in vitro* [114, 144, 145]. Systematically administrating H3R antagonists to activate the histaminergic system can improve relative theta power in the hippocampus while antagonizing H1R can reduce the theta power [86, 130]. The generation of dose-dependent, transient gamma oscillations in CA3 specifically depends on H1R, which is triggered by the activation of histamine receptors [146]. Furthermore, H3R antagonists can rescue reduced gamma oscillations in hippocampal slices from 6-hydroxydopamine (6-OHDA) induced Parkinson’s disease mouse model [147]. This evidence suggests that the histaminergic neuron system has the ability to modulate emotional memory formation.

The details about how histamine acts in emotional memory are still unresolved. In other words, histamine acting through which histamine receptors in which brain regions modulate emotional memory are almost unknown. The underlying characteristics still remain to be determined.

## 7 CLINICAL TRIALS OF CHEMICALS TARGETING HISTAMINERGIC RECEPTORS

Several brain disorders have been described to have modulations in the brain histaminergic system, which may play a significant role in their pathophysiology. For instance, individuals with Alzheimer's disease (AD) exhibit a decrease in the number of HDC-positive neurons in the TMN, but there is no alteration in HDC mRNA expression in the TMN [148, 149]. Additionally, various studies have reported reduced levels of histamine in the hippocampus, frontal cortex, temporal cortex, occipital cortex, and caudate nucleus using different methods [150]. A radioligand for H1R revealed that the binding potential of H1R is reduced in the frontal and temporal areas of patients with AD, and H3R binding in the frontal cortex correlates with dementia severity, while H3R binding is not different between the brains of individuals with AD and age-matched controls [151]. Nonetheless, the binding of H2R remains unchanged in the prefrontal cortex of patients with AD [152].

Given that emotional dysregulation and memory defects usually occur simultaneously in several brain disorders, the dual role of histamine in emotion and memory may enable itself to be a potential target for many cognitive dysfunctions. To explore this hypothesis, several clinical studies have assessed whether the histaminergic system can modulate cognitive processes in humans (Table **3**). Some of the studies investigate the effects of H1R antagonist and found that some of which doesn’t produce any deleterious effect on cognitive and psychometric functions, while some of them can impair memory or attention. According to certain researchers, there is a correlation between the quantity of antihistamine that infiltrates the brain and the severity of clinical symptoms [153-156]. Regarding H2R antagonists, there have been significantly fewer clinical trials conducted. One of these trials discovered that nizatidine did not have any impact on cognitive outcome measures throughout the one-year duration of the study [157]. Finally, the H3R inverse agonist MK-0249 or CEP-26041 was not effective in improving cognitive function in Alzheimer’s disease patients or patients with schizophrenia [158-160]. Betahistine did not have any impact on working memory and learning performance in one study. In another study, it was found that while betahistine can increase the accuracy of individuals who initially performed poorly under placebo treatment and enhance the accuracy of difficult tasks, it can also lead to a decrease in accuracy among those who had better initial performance [133, 161]. It can be inferred from these clinical trials that certain drugs related to histamine may have an impact on regulating learning and memory functions; however, more focused research is required to confirm this. The role of histamine-related drugs in emotional memory could be explored in future clinical trials in conjunction with imaging and psychology-related tools such as fMRI. Furthermore, it is interesting to figure out whether histamine-related drugs can promote emotional memory better in patients with emotion disorders compared to healthy volunteers. Clinical research can group patients according to their emotional state by using the Positive and Negative schedule [162], emotion discrimination test, Hamilton Rating scale for Anxiety (HAM-A), Hamilton Depression Rating Scale (HAM-D) *etc*. The drug's effectiveness can be assessed based on this model of grouping.

## 8 PROSPECT FOR THE STUDY OF HISTAMINE IN THE EMOTIONAL MEMORY

### 8.1 Diverse Functions of Histamine in Emotional Salience or Valence Processing and Encoding

The evidence so far has supported the notion that both reward stimulus and stress stimulus can activate histaminergic neurons. However, activating histaminergic neurons plays a role in reward inhibition and worsens the stress state. Thus, to influence appetitive and aversive behaviors, brain histamine acts in concert with and as a complement to both primary reward and punishment systems. As for the evidence of histamine’s role in salience encoding, neuronal histamine has been proposed to be a sensitive indicator of different kinds of stress and is involved in the neuropathology of emotional disorders for emotionally arousing events that activate histaminergic neurons. Thus, histamine probably interplays with both emotion salience and valence in emotional memory modulation, which requires more experimental evidence.

More experiments are needed in the future to test our suspicion: firstly, no histamine has been reported to respond to and encode both salience and valence in the same experiment, and previous inferences have been drawn by comparing results between reports, so there is an urgent need to examine histamine responses to both salience and valence in the same experimental paradigm. Using the principles of the Pavlovian conditioning paradigm, we can couple emotional unconditioned stimuli of different salience and valence with neutral conditioned stimuli of the same type but not identical and recognizable to each other. Neutral conditioning stimuli can be different frequencies of sound signals, different smells or different visual cues. Unconditioned stimuli can be divided into positive and negative stimuli. Positive stimuli can be the administration of different volumes of water, different concentrations of sugar water, *etc*., under dehydration preconditioning, while negative stimuli can be air puffs, electrical stimuli of different intensities, *etc*. The response of the histaminergic nervous system to salience or valence is investigated by recording changes in single-cell calcium or electrical signals in histaminergic neurons during behavior, by recording changes in histamine concentrations in downstream brain regions with histamine probes, or by recording changes in calcium signals in downstream brain regions with histaminergic neuronal axons. In innate valence encoding, the signal will be opposite for oppositely valenced unconditioned stimuli but not respond to conditioned stimuli. Valence-encoding neurons typically maintain responsiveness to the US while the US contains affective information but can acquire responsiveness to the CS (acquired valence) so that the CS can evoke an affective response. Regardless of the valence of the stimulus, salience-encoding neurons should respond in the same direction. These cells can have a response to the CS but not to the US. Alternatively, salience-encoding neurons may initially respond to the US. However, this response rapidly diminishes as salience is transferred to the predictive CS. Since histaminergic neurons project to almost all brain regions and their function is complex, after determining the function of the histaminergic nervous system for either valence or salience, it is possible to classify histaminergic neurons that encode different salience and valence of stimuli, such as positive or negative, by means of cFos labeling, and to investigate whether there are subpopulations of histaminergic neurons that encode one or more of these features. It is also possible to use opto-GPCRs (optoH1R/ H2R/H3R) to modulate H1/H2/H3 receptors in specific downstream brain regions of histaminergic neurons to clarify the neural circuits mechanisms encoded by the histaminergic nervous system in salience and valence [163]. Furthermore, using spatial transcriptome and single-cell sequencing techniques to analyze different subpopulations to find potential drug targets for emotional memory disorders [164, 165].

### 8.2 The Precise Role of Histamine and Histamine Receptors in Different Emotional Memory Processes

**Fig. (3):**
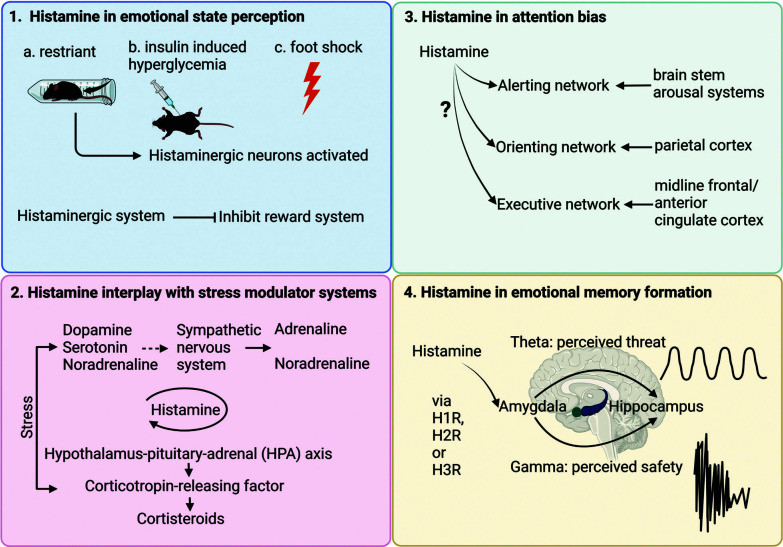
Schematics about how histamine works on emotional memory. (**1**) evidence to date implies that histamine can modulate emotional state perception. Histaminergic neurons can be activated by restraint stress, insulin-induced hyperglycemia stress and foot shock stress. And histaminergic system can interact with a reward system, which might influence positive memory. (**2**) histamine can interplay with stress systems which include monoamines and HPA axis activation. Histamine can activate stress modulator systems and influence emotional memory. (**3**) attention systems include alerting network, orienting network and executive network. The exact role of histamine in attention regulation remains to be elucidated. (**4**) pharmacologic evidence showed that histaminergic systems can influence emotional memory formation innervating amygdala and hippocampus. These processes might involve changes in local field potential changes in theta and gamma oscillation *via* H1R, H2R or H3R.

Numerous studies have emphasized the functions of histaminergic projections to BLA, hippocampus, basal forebrain, and cerebellar vermis in aversive memory while to the brain reward system, including the nucleus accumbens, in reward-related memory. However, histaminergic projections to the hippocampus and PRh can also modulate neutral memory, like NOR. There are supposed to be a number of differences among the roles of histamine in modulating aversive, neutral, and positive memories, such as the different neural circuits underlying different emotional memories. Histaminergic projections are extensively distributed in various parts of the brain, such as the cortex, hippocampus, amygdala, striatum, basal forebrain, and cerebellum. However, histamine receptors are not evenly distributed all over the brain, and the histaminergic system can influence other neurotransmitters through heteroreceptors histamine H3R. Thus, the heterogeneity of the histaminergic systems allows for the modulation of different emotional memories through the activation of various receptors and brain regions. Emotional memory has complex formation processes that differ from neutral and mundane memory. More fine-grained behavioral studies can be designed to determine the function of the histaminergic nervous system in the encoding of emotional memory and its specific stages (Fig. **3**). Further, the association between histamine release and network oscillation or the rhythmics of specific frequency electrical signals within specific brain regions can be recorded in real-time. As well as using genetics and optogenetics to explore the role of instinctive histamine receptors in specific emotional memory processes. Using TRAP-related genetics to explore the function of histamine in emotional memory-related networks in engram cells. Finally, imaging tools such as fMRI and related cognitive experiments can be used with human subjects to explore whether interventions in the histaminergic nervous system affect features specific to emotional memory, such as attentional bias during emotional memory encoding. Future clinical studies evaluating the therapeutic effects of histamine-related drugs in memory disorders can also take into account the emotional state of the patient when assessing their efficacy.

## CONCLUSION

In summary, histamine is an important responsive neuromodulator to emotional states such as stress, anxiety or reward-related signals. Thus, we infer that histamine has the ability to encode the emotional valence and salience of stimuli and histamine plays an important role in emotional memory consolidation and retrieval, which may be related to associative processes linking sensory stimuli and between the CS and the value of US. Nevertheless, histamine's more specific role in emotional learning and memory remains unclear. The use of cutting-edge technology, the dissection of histaminergic circuits and the spatiotemporally selective manipulation of histamine receptors will help to address these issues and provide insight into clinical strategies for the treatment of emotion and memory disorders.

## Figures and Tables

**Table 1:** Main differences between negative memory and positive memory characteristics.

**The Characteristics of Memories**	**Negative Memory**	**Positive Memory**	**References**
Recollection	++	+	[166]
Attentional capture effects	++	+	[167]
Retrieval	More automatic	More vividness and reexperiencing the original event come to mind more frequently.	[168]
Associations	More sensory specificity, with item-specific details	More of the conceptual framework or gist	[3]
Fade effects	The effect of negative memories tends to fade	The effect of positive memories can keep longer	[33]
Connections	Engage sensory regions may be associated with enhanced amygdala-binding and reduced hippocampal-binding	Engage prefrontal regions, both amygdala-binding and hippocampal-binding	[34-36]

**Note:** “++” indicates a stronger degree, while “+” indicates normal degree.

**Table 2:** Main findings regarding the roles of histamine receptors on learning and memory.

**Roles**	**Receptors**	**Localizations**	**Evidence**	**References**
Fear memory consolidation	H1R	Postsynaptic	(1) Antagonize H1R directly has no effect; (2) Using an H1R antagonist and thioperamide simultaneously can’t block the effects of THIO.	[66, 111, 112, 116, 118, 121, 122, 124, 169, 170]
		In NBM, postsynaptic		
		In CA1, postsynaptic		
		In cerebellar vermis, postsynaptic		
	H2R	Postsynaptic	(1) Antagonize H2R directly has no effect or impairs memory. On the contrary, activating H2R can improve memory; (2) Using an H2R antagonist and thioperamide simultaneously can block the effects of THIO.	
		In NBM, postsynaptic		
		In CA1, postsynaptic		
		In cerebellar vermis, postsynaptic		
		In septum, postsynaptic		
	H3R	Presynaptic	(1) When H3R were postsynaptic, activate H3R promote memory *vice versa*; (2) In BLA, activating H3R can increase Ach release, thus facilitating consolidation.	
		In NBM, presynaptic		
		In CA1, presynaptic		
		In cerebellar vermis, presynaptic		
		In septum, postsynaptic?		
		In BLA, postsynaptic?		
Fear memory retrieval	H1R	postsynaptic	(1) Inhibit H1R can inhibit memory retrieval.	[108, 111, 114-118]
		In CA1, postsynaptic		
	H2R	In CA1, postsynaptic	(1) Active H2R was ineffective to restore fear memory.	
		In BLA, postsynaptic		
		In vmPFC, postsynaptic		
	H3R	Presynaptic?	Antagonized H3R can improve memory retrieval.	
Morris-water maze	H1R	Postsynaptic	(1) In H1RKO mice, memory↓; (2) In HDC-/- mice, memory ↓ or ↑; (3) Both inhibit or activate H3R can reverse the memory deficit in scopolamine-induced memory deficit; (4) Inhibit H2R and decrease spatial learning and memory.	[58, 60, 145, 171-178]
		In MEC, postsynaptic		
	H2R	Postsynaptic		
	H3R	presynaptic		
		In MEC, presynaptic		
Barnes-maze	H1R	postsynaptic	(1) Histamine improved working memory and reference memory deficits induced by scopolamine; (2) Antagonize H1R or H2R abolish the ameliorative effects of histamine; (3) Block H3R can reverse the deficit or improve the deficit.	[57, 59-61, 68, 176, 179-182]
		In the hippocampus, postsynaptic		
	H2R	postsynaptic		
		In the hippocampus, postsynaptic		
	H3R	presynaptic		
		In the hippocampus, presynaptic/postsynaptic?		
Novel object recognition	H1R	postsynaptic	(1) Block H3R can alleviate memory deficit in amnesia models; (2) In CA1, inhibit H1R, H2R or activate H3R at the consolidation early stage can prevent memory recall; (3) In PRh, histamine can enhance memory retrieval through H2R or H3R and facilitate forgotten memory retrieval.	[114, 115, 118, 132, 133, 183, 184]
		In CA1, postsynaptic		
		in PRh, postsynaptic		
	H2R	postsynaptic		
		In CA1, postsynaptic		
		In PRh, postsynaptic		
	H3R	presynaptic		
		In CA1, presynaptic		
		In PRh, presynaptic		
Object location recognition memory	H1R	In sMEC, postsynaptic	Activating H1R and H3R in sMEC can promote OLM.	[182, 185, 186]
	H2R	In sMEC, postsynaptic		
	H3R	In sMEC, presynaptic		

**Table 3:** Main clinical studies regarding the impacts of histamine-related compounds on learning and memory.

**Drug**	**Target**	**Population**	**Characteristics**	**Procedure**	**Treatment**	**Effects on Memory**	**References**
Betahistine	H1 &H3	Healthy right-handed volunteers	18-55 years N = ♀8♂8	N-back working memory task (working memory), visuospatial paired associates learning task (learning)	Placebo + betahistine 48 mg (n = 16)	No betahistine-induced changes in brain activity were found in these networks.	[161]
Betahistine	H3	Healthy adults	N = 38 subjects	Paired-associate learning task and object recognition behavior task	Training, day/ 10 108 mg self-comparison	Betahistine enhanced the correct rate of subjects who had poor performance under placebo treatment; enhanced the correct rate of subjects with middle-range IQ; improved the correct rate for difficult items; reduced the correct rate of subjects who had better performance under placebo treatment and subjects with low and high IQ, and it reduced the rate for easy items.	[133]
MK-0249	H3 inverse agonist	Patients with schizophrenia who were clinically stable experienced no more than mild to moderate overall symptoms (PANSS score total 36-75), and were taking a stable dose of antipsychotic.	21-55 years N = ♀15♂40 35 white, 20 Asian		2-period (4 weeks per period) placebo + MK-0249 10 mg (n = ?)	MK-0249 10 mg once daily was not superior to a placebo in the treatment of cognitive impairment in patients with schizophrenia after 4 weeks. (ClinicalTrials.gov: NCT00506077)	[158]
MK-0249	H3 inverse agonist	MMSE inclusive score 18-26 (mild-to-moderate), AD patients	>55 years ♀79♂65 139 white	Word List Learning-Selective Reminding, Word-List Learning-Delayed Recall, Word List Learning-Delayed Recognition, Simple Reaction Time, Choice Reaction Time, Visual Memory, and Object Naming.	4 weeks placebo + MK-0249 5 mg (n = 144)	MK-0249 5 mg once daily over 4 weeks was not effective in improving cognitive function in mild to moderate AD patients who were on concomitant symptomatic AD treatment. (ClinicalTrials.gov trial registration, NCT00420420).	[159]
	H3R	Right-handed healthy adults	N = 10 ♂ 25 ± 4years	N-back task to assess neural activities related to working memory using fMRI&PET.	-	Higher neural activity of working memory was associated with lower H3 receptor density in the right dorsolateral prefrontal cortex.	[187]
GSK239512	H3R antagonist	Patients with mild to moderate Alzheimer’s disease	N = 71 ♂129♀ 50-97years	Cognitive function was primarily assessed using a neuropsychological test battery (NTB)	2w: run-in period; 4w: titration from 10 μg/day- 80 μg/ day; 12w: maintenance phase; 2w: follow-up phase	GSK239512, at doses up to 80μg/day, improved Episodic Memory in patients with mild to-moderate AD. However, no improvements were observed on Executive Function/ Working Memory or other domains of cognition.	[188]
CEP-26401	H3R antagonist	Healthy volunteers	N = 40	SWM, rapid visual information processing, stop signal task, paired associate learning, visual, verbal learning task, maze learning, N-back, Stroop choice reaction time.	CEP-26401 5, 25 or 125 μg + placebo	The H3R antagonist CEP‐26401 positively influenced spatial working memory with the best effect at the lowest dose. CEP-26401 did not have any positive effects on cognitive tests.	[160]
d-chlorpheni-ramine	H1 antagonist	Healthy adults	20-27 years N = ♂43	Assessment of changes in task performance and subjective sleepiness (n = 8); H1R occupancy (n = 20); rCBF measurement (n = 12); global measurement (n = 3).	2 mg d-chlor-pheniramine + placebo	↑ in the right prefrontal and anterior cingulate cortices; ↓ in the left temporal and frontal cortices and midbrain after the treatment of d-chlorpheniramine. The results indicated that the attention system of the human brain could be altered by therapeutic doses of H1R antagonists.	[189]
Levocetirizine, diphenhydramine	H1R antagonist	Healthy volunteers. Subjects were trained beforehand to attain baseline performance in the test battery and to become familiar with test procedures.	N = 24 ♂24♀ 23.3 ± 2.2 years, weight 69.8 ± 10.3 kg, height 1.78 ± 0.09 m	A word-learning test, the Sternberg memory scanning test, a tracking test (easy and hard version), a divided attention test (tracking and memory scanning simultaneously)	Acute administration 1 day; sub-chronic administration 4 days 3 hours before the start of the lab test battery. 5 mg levocetirizine or 50 mg diphenhydramine	The results show that memory, attention, and tracking performance are unaffected after acute and subchronic administration of levocetirizine (5 mg), whereas diphenhydramine (50 mg) significantly affected divided attention and tracking after acute administration.	[190]
Bilastine, cetirizine	H1R antagonist	Healthy volunteers above 18 years with normal BMI and non-smokers, no history of associated chronic illnesses and not being on medication on a permanent basis.	N = 17♂16♀ 37.06 ± 9.09 years	-	Bilastine, cetirizine and placebo, after a minimum washout time of seven days.	Bilastine did not impair the tested abilities in comparison with the control groups, either at ground level or hypobaric hypoxia. Administration of cetirizine increased the number of errors at ground level. At the simulated altitude, already impaired results were additionally demonstrated with regard to the distributive attention test.	[153]
Cetirizine 10 mg, loratadine 10 mg, diphenhydramine 50 mg, chlorpheniramine 8 mg	H1R antagonist	Subjects were recruited in response to an advertisement and were eligible to participate if they were >65 years of age, enjoyed good physical and mental health, lived independently in the community, and did not require any medication on a regular basis.	N = 17♂16♀ 71 ± 5 years	Cognitive processing was objectively assessed using a change in latency of the P300-event-related potential (P300).	Single-dose outcome measures were recorded before and 2 to 2.5 hours after dosing.	In the elderly, the new H1R antagonists cetirizine and loratadine are less likely to cause adverse central nervous system effects than the old H1R antagonists chlorpheniramine or diphenhydramine, but this requires confirmation using additional objective tests of central nervous system function.	[191]
Rupatadine 10, 20, 40 mg	H1R antagonist and a potent antagonist of the pro-inflammatory lipid mediator	Subjects were healthy who were born in Japan to both Japanese parents and grandparents, lived less than 5 years outside of Japan and who did not have a significant change in lifestyle since leaving Japan.	N = 21♂6♀, 20-45 years, BMI18-25	The Cogtest Battery in this study included rapid visual information processing continuous performance task, reaction time, spatial working memory and visual analog scales.	The subjects were admitted on Day -2 (*i.e*. 2 days before the administration of the first dose), received a placebo on Day -1 and a single dose of rupatadine or placebo on Day 1 followed by once daily doses on Days 2-5.	The therapeutic dose of rupatadine did not show any CNS impairment in any of the cognitive tests.	[154]
Mizolastine 5 mg, 15 mg, 45 mg; terfenadine 60 mg; triprolidine 10 mg	H1R antagonists	Healthy volunteers.	N = 18♂ 18-40 years	Critical flicker fusion, choice reaction time, tracking, Stroop and Sternberg memory tests and assessment of subjective sedation.	Following each dose, subjects performed a series of tests of cognitive function and psychomotor performance at 1, 3, 5, 8 and 24 hours post-dose.	Mizolastine (5 mg, 15 mg) is free from disruptive effects on cognitive function and psychomotor performance, in contrast to terfenadine 60 mg, triprolidine 10 mg and mizolastine 45 mg.	[192]
Levocetirizine 5 mg; diphenhydramine 50 mg	H1R antagonist	Healthy volunteers with no addiction on alcohol or any other objects.	N = 19♂ 18-40 years	Critical flicker fusion, choice reaction time, body sway, learning memory test and subjective assessments of alertness	Receive the drug once daily for 5 consecutive days.	Levocetirizine does not produce any deleterious effect on cognitive and psychometric functions compared with placebo in healthy male volunteers.	[193]
Ebastine10, 20, 30 mg; triprolidine 10 mg	H1R antagonist	-	-	Critical flicker fusion, choice reaction time, tracking, Stroop and Sternberg memory tests, assessment of subjective sedation and subjective evaluation of sleep.	Perform a series of tests of cognitive function and psychomotor performance at 1h, 2h, 3h and 8h post-dose on day 1 and 5	Ebastine, at its recommended therapeutic doses of 10-20 mg, is demonstrably free from impairment on objective aspects of psychomotor and cognitive function in a study where the psychometric assessments were shown to be sensitive to disruptive effects, as evidenced by the action of the positive control, triprolidine 10 mg.	[194]
Diphenhydramine (50, 75, 100 mg), lorazepam (0.5,1.5 mg)	H1R antagonist	Healthy volunteers	N = 6♂6♀ 20-33 years	Subjective assessments of sedation, sleep latencies, digit symbol substitution, choice reaction time, sustained attention and memory recall	Volunteers were studied 1.0 h before and 0.5, 2.0 and 3.5h after drug ingestion.	It is considered that impaired memory is not necessarily associated with sedation and that impairment of memory with drugs that lead to sedation may be affected through neuronal systems independent of those that affect arousal.	[195]
Citerizine 10, 20 mg	H1R antagonist	Healthy volunteers	N = 17 23 ± 2.6 years	Effects on cognition were assessed using tests of word learning, memory scanning, vigilance, divided attention, tracking and visual information processing speed.	Single dose	Cetirizine 10 mg impaired tracking performance, and both doses impaired memory scanning speed. None of the other measures indicated impaired performance.	[155]
dexchlorpheniramine 4 mg, lorazepam 1 mg	H1R antagonist	Healthy volunteers	N = 18 24.2 ± 1.7 years	The battery consisted of a word learning task, an n-back task and subjective alertness and was performed in this order. In addition, objective evaluations of alertness were obtained during word learning task performance.	Single dose	The active control lorazepam impaired episodic- and working memory performance and increased sedation, while dexchlorpheniramine only increased sedation.	[156]
fexofenadine (FEX) and cetirizine (CET)	H1R antagonist	Healthy Japanese volunteers	N = 8 in PET; N = 16 in behavior test 20-28 years	H1R occupancy by antihistamines was examined by PET with ^11^C-doxepin; Subjective sleepiness was measured using the Stanford Sleepiness Scale (SSS) and psychomotor performance was examined by a tachistoscope testing system	-	Histamine and H1Rs are involved in maintaining arousal and cognition in humans, and that the severity of clinical symptoms is correlated to the amount of antihistamine that penetrated into the brain.	[196]
Nizatidine 75 mg	H2R antagonist	Participants with AD were identified by the Cache County Study on Memory in Aging. More than 97% of participants provided a buccal scraping for APOE genotyping.	N = 51♂♀ 67-96 years	Using tests from the CERAD battery and additional measures of visuospatial memory, verbal memory, and verbal fluency	Patients receive tablets twice daily. Cognitive outcomes were assessed at baseline, six and twelve months after enrollment.	Intention-to-treat and compliance-based analyses showed no effect of nizatidine on any of the cognitive outcome measures over the one-year study interval.	[157]
Related review	[197, 198]						

## LIST OF ABBREVIATIONS

AD: Alzheimer's Disease
BLA: Basolateral Amygdala
CNS: Central Nervous System
CPP: Conditioned Place Preference
CRH: Corticotropin-releasing Hormone
CTA: Conditioned Taste Aversion
EPM: Elevated Plus Maze
H1R: Histamine H1 Receptor
H2R: Histamine H2 Receptor
H3R: Histamine H3 Receptor
HDC: Histidine Decarboxylase
HPA: Hypothalamic-pituitary-adrenal
mPFC: Medial Prefrontal Cortex
NBM: Nucleus Basalis of Meynert
PD: Parkinson's Disease
PFC: Prefrontal Cortex
PRh: Perirhinal Cortex
PTSD: Post-traumatic Stress Disorder
PVN: Paraventricular Nucleus of the Hypothalamus
PVT: Paraventricular Thalamus
RAMH: R-(−)-α-methylhistamine Dihydrobromide
sMEC: Superficial Medial Entorhinal Cortex
αFMH: α-fluoromethyl l-histidine
